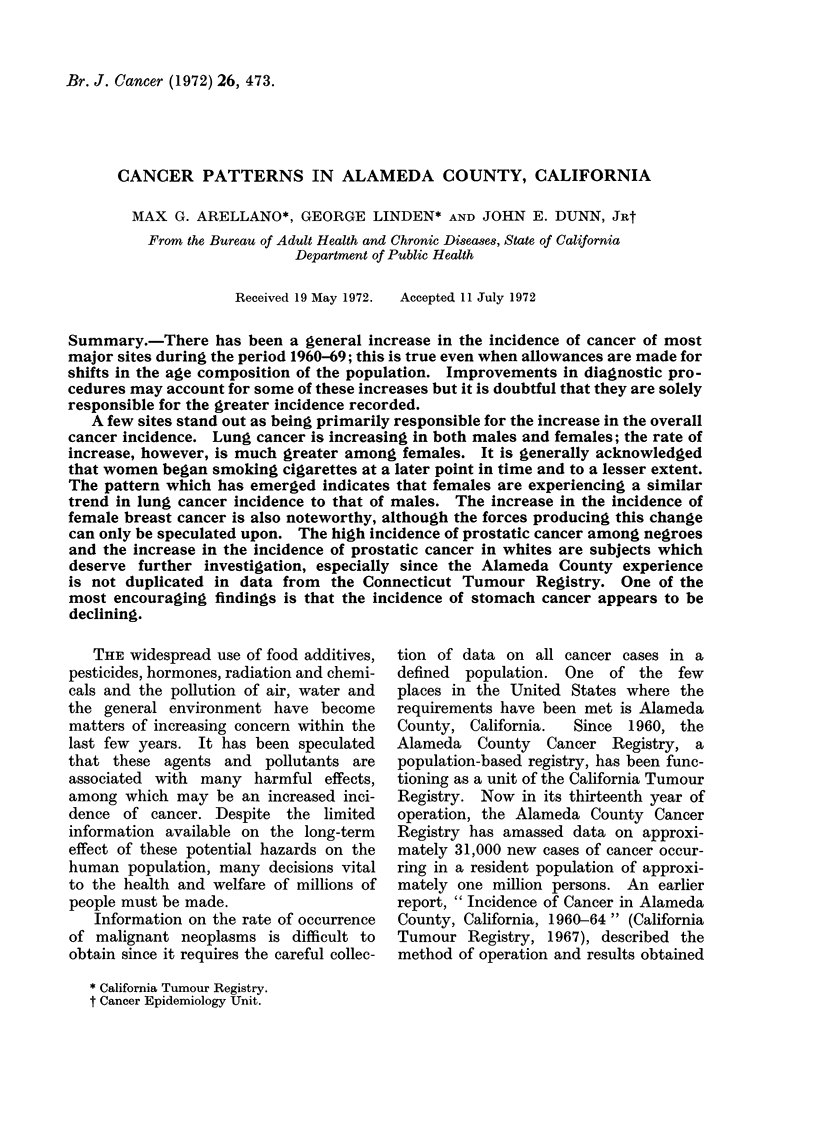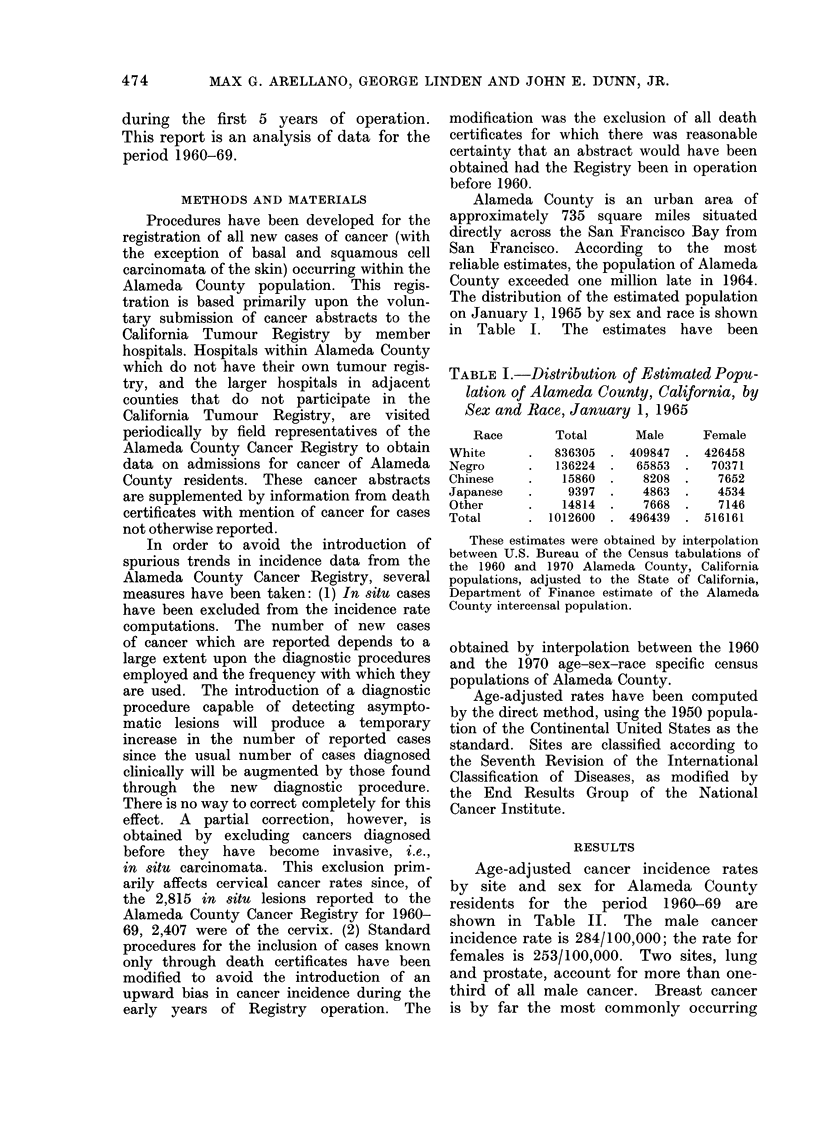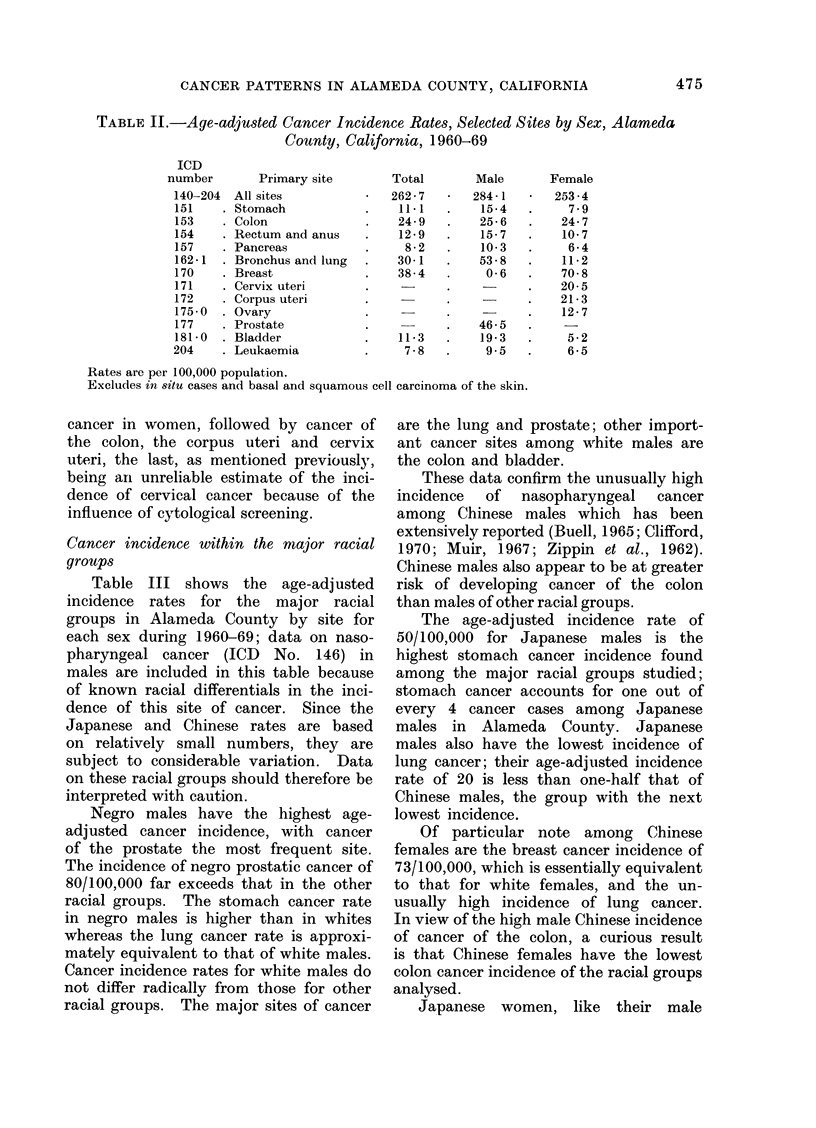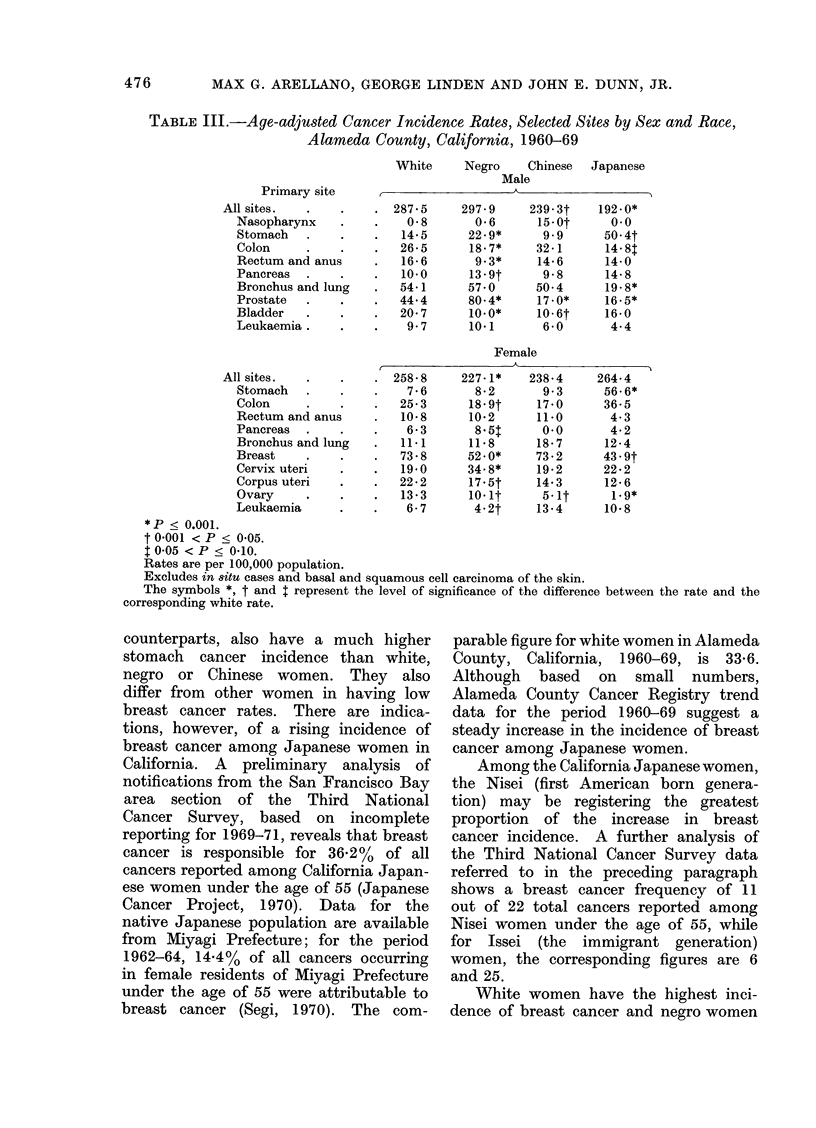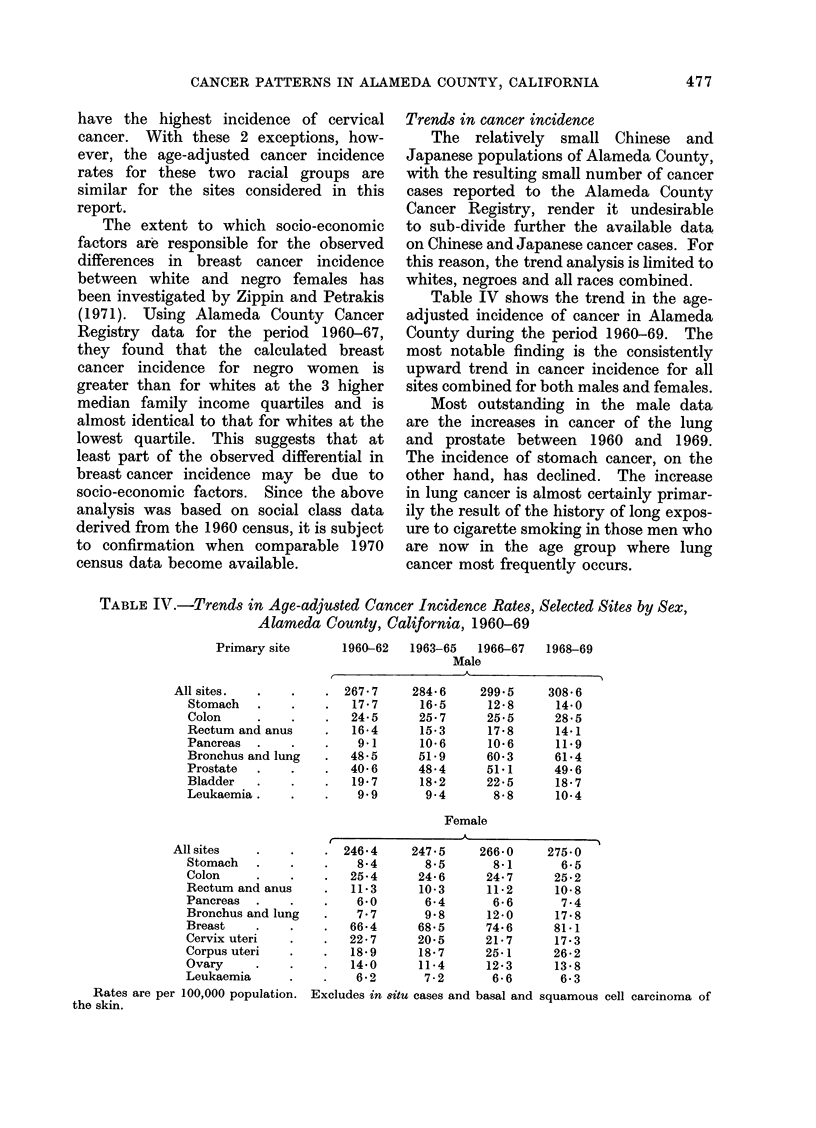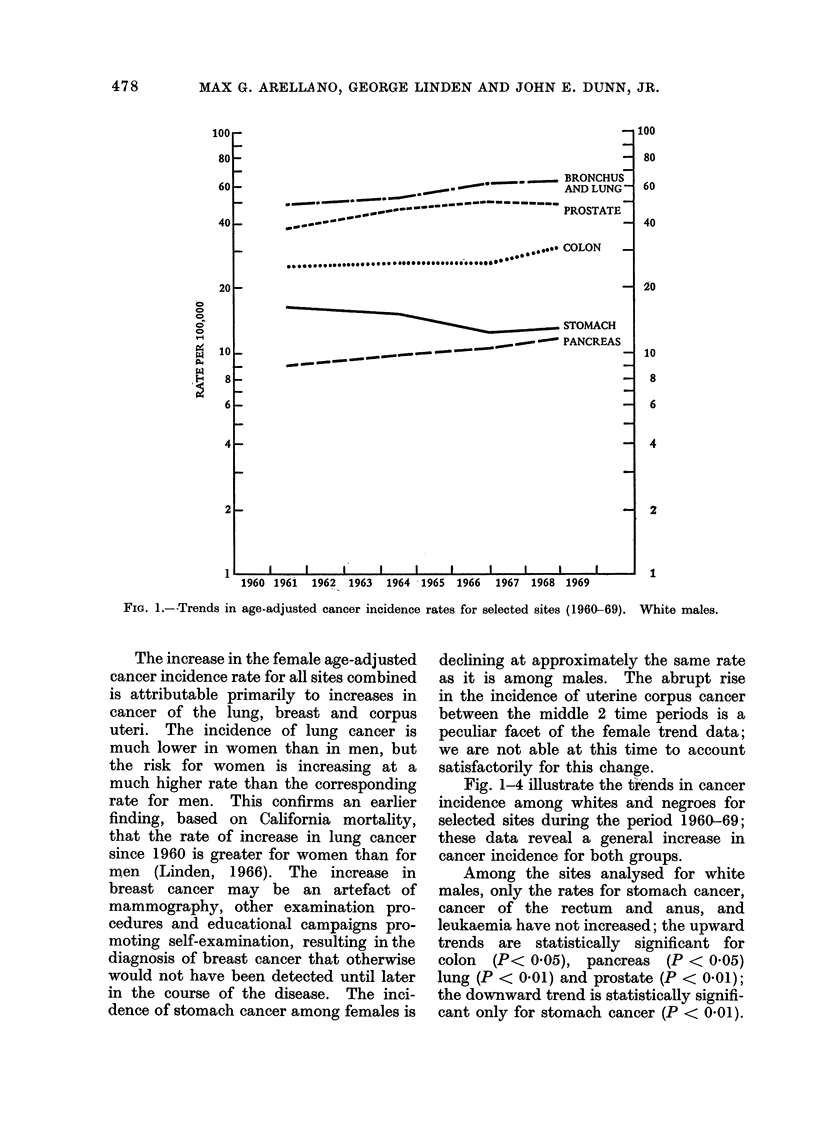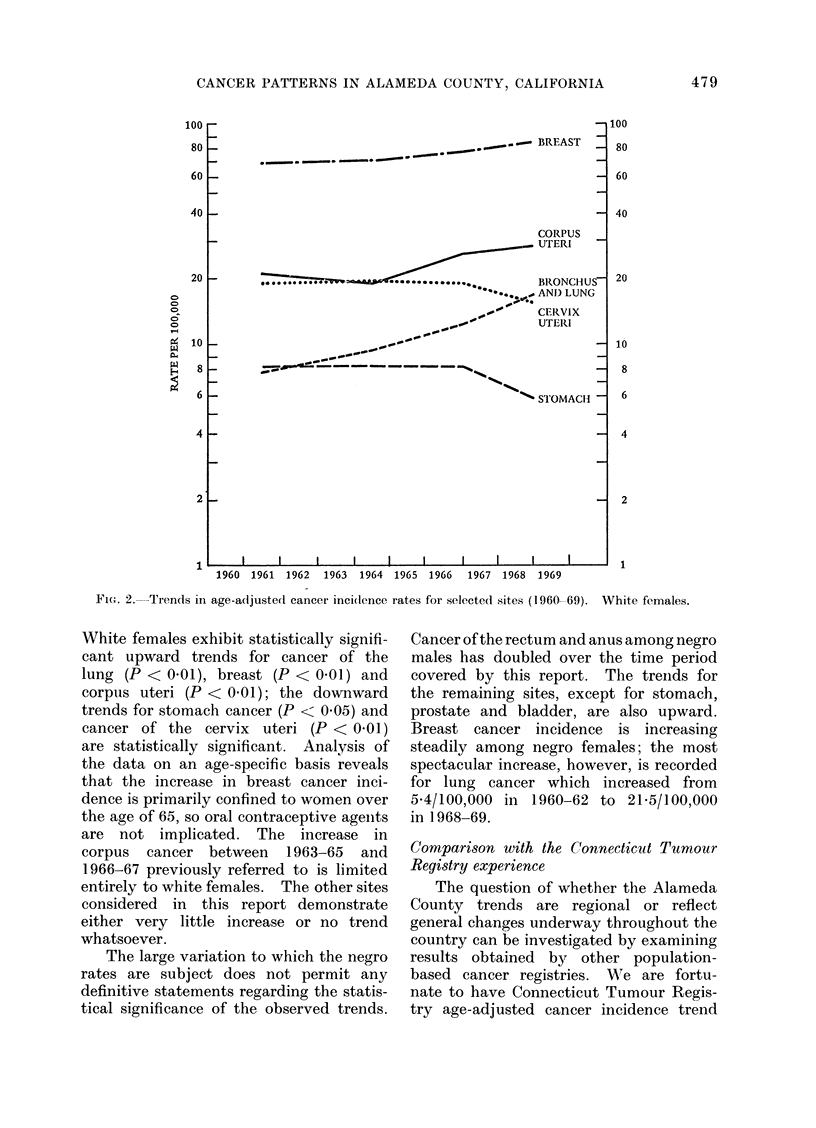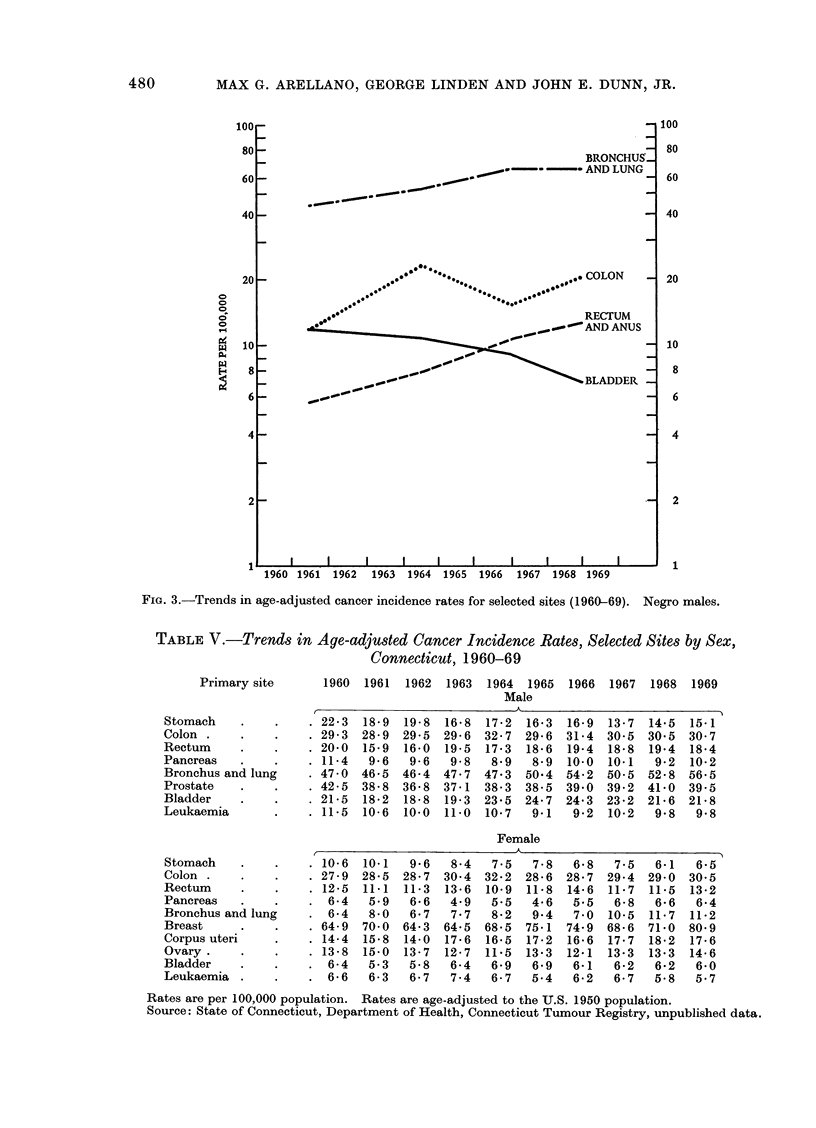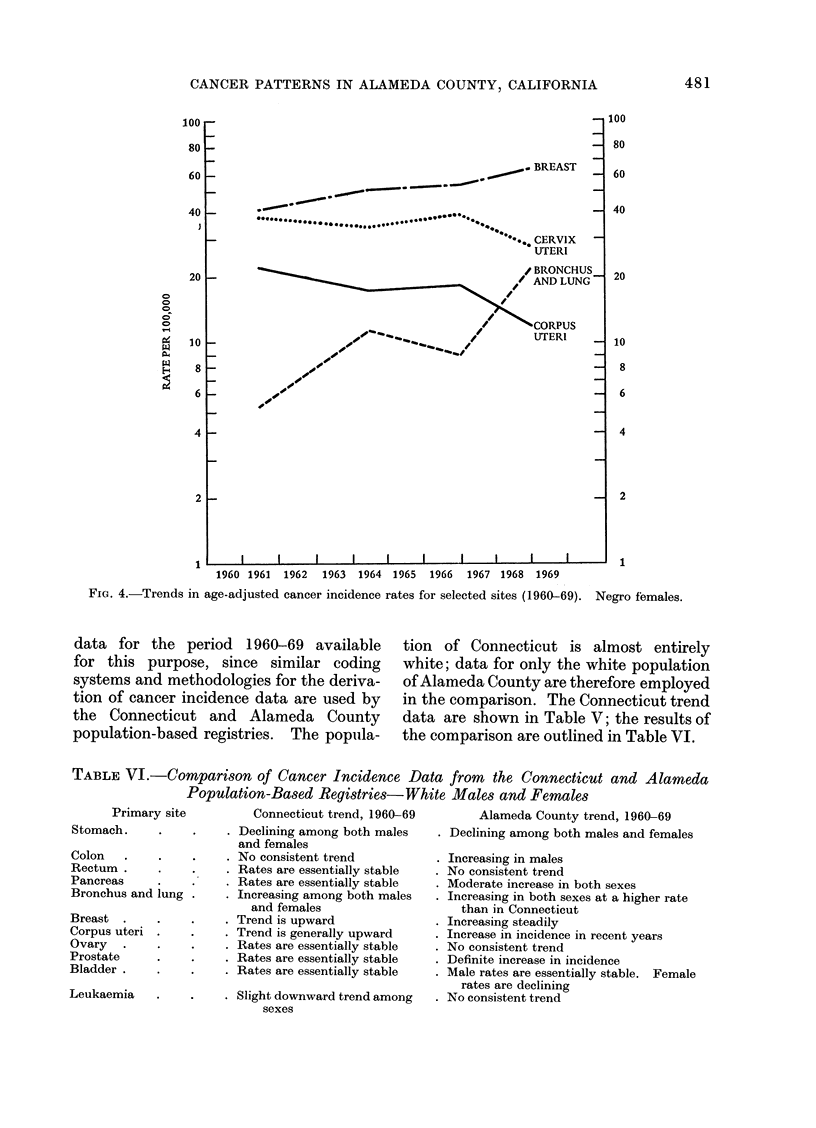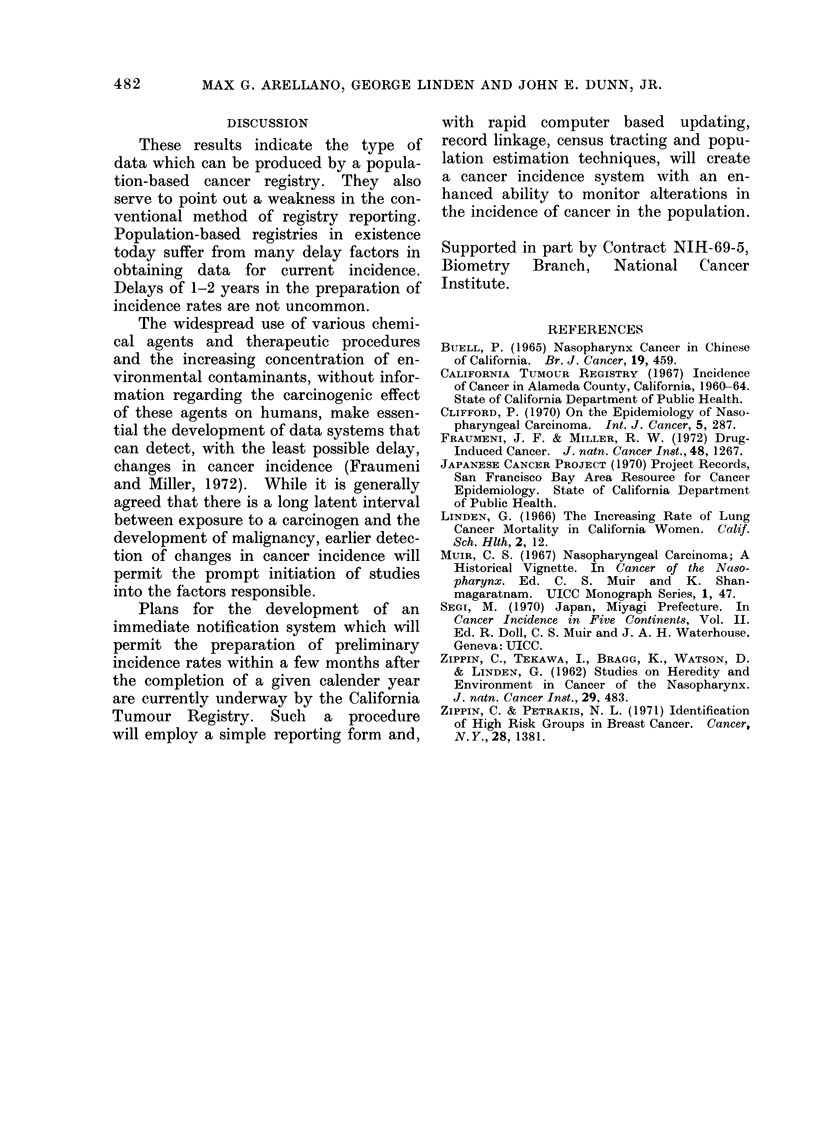# Cancer Patterns in Alameda County, California

**DOI:** 10.1038/bjc.1972.65

**Published:** 1972-12

**Authors:** Max G. Arellano, George Linden, John E. Dunn

## Abstract

There has been a general increase in the incidence of cancer of most major sites during the period 1960-69; this is true even when allowances are made for shifts in the age composition of the population. Improvements in diagnostic procedures may account for some of these increases but it is doubtful that they are solely responsible for the greater incidence recorded.

A few sites stand out as being primarily responsible for the increase in the overall cancer incidence. Lung cancer is increasing in both males and females; the rate of increase, however, is much greater among females. It is generally acknowledged that women began smoking cigarettes at a later point in time and to a lesser extent. The pattern which has emerged indicates that females are experiencing a similar trend in lung cancer incidence to that of males. The increase in the incidence of female breast cancer is also noteworthy, although the forces producing this change can only be speculated upon. The high incidence of prostatic cancer among negroes and the increase in the incidence of prostatic cancer in whites are subjects which deserve further investigation, especially since the Alameda County experience is not duplicated in data from the Connecticut Tumour Registry. One of the most encouraging findings is that the incidence of stomach cancer appears to be declining.


*Br. J. Cancer* (1972) **26**, 473.

### CANCER PATTERNS IN ALAMEDA COUNTY, CALIFORNIA

MAX G. ARELLANO*, GEORGE LINDEN* AND JOHN E. DUNN, JR†

*From the Bureau of Adult Health and Chronic Diseases, State of California Department of Public Health*

Received 19 May 1972. Accepted 11 July 1972

**Summary.—There has been a general increase in the incidence of cancer of most major sites during the period 1960–69; this is true even when allowances are made for shifts in the age composition of the population. Improvements in diagnostic procedures may account for some of these increases but it is doubtful that they are solely responsible for the greater incidence recorded.**

**A few sites stand out as being primarily responsible for the increase in the overall cancer incidence. Lung cancer is increasing in both males and females; the rate of increase, however, is much greater among females. It is generally acknowledged that women began smoking cigarettes at a later point in time and to a lesser extent. The pattern which has emerged indicates that females are experiencing a similar trend in lung cancer incidence to that of males. The increase in the incidence of female breast cancer is also noteworthy, although the forces producing this change can only be speculated upon. The high incidence of prostatic cancer among negroes and the increase in the incidence of prostatic cancer in whites are subjects which deserve further investigation, especially since the Alameda County experience is not duplicated in data from the Connecticut Tumour Registry. One of the most encouraging findings is that the incidence of stomach cancer appears to be declining.**

THE widespread use of food additives, pesticides, hormones, radiation and chemicals and the pollution of air, water and the general environment have become matters of increasing concern within the last few years. It has been speculated that these agents and pollutants are associated with many harmful effects, among which may be an increased incidence of cancer. Despite the limited information available on the long-term effect of these potential hazards on the human population, many decisions vital to the health and welfare of millions of people must be made.

Information on the rate of occurrence of malignant neoplasms is difficult to obtain since it requires the careful collection of data on all cancer cases in a defined population. One of the few places in the United States where the requirements have been met is Alameda County, California. Since 1960, the Alameda County Cancer Registry, a population-based registry, has been functioning as a unit of the California Tumour Registry. Now in its thirteenth year of operation, the Alameda County Cancer Registry has amassed data on approximately 31,000 new cases of cancer occurring in a resident population of approximately one million persons. An earlier report, " Incidence of Cancer in Alameda County, California, 1960–64 " (California Tumour Registry, 1967), described the method of operation and results obtained

* California Tumour Registry.
† Cancer Epidemiology Unit.

during the first 5 years of operation. This report is an analysis of data for the period 1960–69.

### METHODS AND MATERIALS

Procedures have been developed for the registration of all new cases of cancer (with the exception of basal and squamous cell carcinomata of the skin) occurring within the Alameda County population. This registration is based primarily upon the voluntary submission of cancer abstracts to the California Tumour Registry by member hospitals. Hospitals within Alameda County which do not have their own tumour registry, and the larger hospitals in adjacent counties that do not participate in the California Tumour Registry, are visited periodically by field representatives of the Alameda County Cancer Registry to obtain data on admissions for cancer of Alameda County residents. These cancer abstracts are supplemented by information from death certificates with mention of cancer for cases not otherwise reported.

In order to avoid the introduction of spurious trends in incidence data from the Alameda County Cancer Registry, several measures have been taken: (1) *In situ* cases have been excluded from the incidence rate computations. The number of new cases of cancer which are reported depends to a large extent upon the diagnostic procedures employed and the frequency with which they are used. The introduction of a diagnostic procedure capable of detecting asymptomatic lesions will produce a temporary increase in the number of reported cases since the usual number of cases diagnosed clinically will be augmented by those found through the new diagnostic procedure. There is no way to correct completely for this effect. A partial correction, however, is obtained by excluding cancers diagnosed before they have become invasive, *i.e.*, *in situ* carcinomata. This exclusion primarily affects cervical cancer rates since, of the 2,815 *in situ* lesions reported to the Alameda County Cancer Registry for 1960–69, 2,407 were of the cervix. (2) Standard procedures for the inclusion of cases known only through death certificates have been modified to avoid the introduction of an upward bias in cancer incidence during the early years of Registry operation. The modification was the exclusion of all death certificates for which there was reasonable certainty that an abstract would have been obtained had the Registry been in operation before 1960.

Alameda County is an urban area of approximately 735 square miles situated directly across the San Francisco Bay from San Francisco. According to the most reliable estimates, the population of Alameda County exceeded one million late in 1964. The distribution of the estimated population on January 1, 1965 by sex and race is shown in Table I. The estimates have been obtained by interpolation between the 1960 and the 1970 age–sex–race specific census populations of Alameda County.

TABLE I.—*Distribution of Estimated Population of Alameda County, California, by Sex and Race, January* 1, 1965

| Race | Total | Male | Female |
|---|---|---|---|
| White | 836305 | 409847 | 426458 |
| Negro | 136224 | 65853 | 70371 |
| Chinese | 15860 | 8208 | 7652 |
| Japanese | 9397 | 4863 | 4534 |
| Other | 14814 | 7668 | 7146 |
| Total | 1012600 | 496439 | 516161 |

These estimates were obtained by interpolation between U.S. Bureau of the Census tabulations of the 1960 and 1970 Alameda County, California populations, adjusted to the State of California, Department of Finance estimate of the Alameda County intercensal population.

Age-adjusted rates have been computed by the direct method, using the 1950 population of the Continental United States as the standard. Sites are classified according to the Seventh Revision of the International Classification of Diseases, as modified by the End Results Group of the National Cancer Institute.

### RESULTS

Age-adjusted cancer incidence rates by site and sex for Alameda County residents for the period 1960–69 are shown in Table II. The male cancer incidence rate is 284/100,000; the rate for females is 253/100,000. Two sites, lung and prostate, account for more than one-third of all male cancer. Breast cancer is by far the most commonly occurring

TABLE II.—*Age-adjusted Cancer Incidence Rates, Selected Sites by Sex, Alameda County, California,* 1960–69

| ICD number | Primary site | Total | Male | Female |
|---|---|---|---|---|
| 140–204 | All sites | 262·7 | 284·1 | 253·4 |
| 151 | Stomach | 11·1 | 15·4 | 7·9 |
| 153 | Colon | 24·9 | 25·6 | 24·7 |
| 154 | Rectum and anus | 12·9 | 15·7 | 10·7 |
| 157 | Pancreas | 8·2 | 10·3 | 6·4 |
| 162·1 | Bronchus and lung | 30·1 | 53·8 | 11·2 |
| 170 | Breast | 38·4 | 0·6 | 70·8 |
| 171 | Cervix uteri | — | — | 20·5 |
| 172 | Corpus uteri | — | — | 21·3 |
| 175·0 | Ovary | — | — | 12·7 |
| 177 | Prostate | — | 46·5 | — |
| 181·0 | Bladder | 11·3 | 19·3 | 5·2 |
| 204 | Leukaemia | 7·8 | 9·5 | 6·5 |

Rates are per 100,000 population.
Excludes *in situ* cases and basal and squamous cell carcinoma of the skin.

cancer in women, followed by cancer of the colon, the corpus uteri and cervix uteri, the last, as mentioned previously, being an unreliable estimate of the incidence of cervical cancer because of the influence of cytological screening.

*Cancer incidence within the major racial groups*

Table III shows the age-adjusted incidence rates for the major racial groups in Alameda County by site for each sex during 1960–69; data on nasopharyngeal cancer (ICD No. 146) in males are included in this table because of known racial differentials in the incidence of this site of cancer. Since the Japanese and Chinese rates are based on relatively small numbers, they are subject to considerable variation. Data on these racial groups should therefore be interpreted with caution.

Negro males have the highest age-adjusted cancer incidence, with cancer of the prostate the most frequent site. The incidence of negro prostatic cancer of 80/100,000 far exceeds that in the other racial groups. The stomach cancer rate in negro males is higher than in whites whereas the lung cancer rate is approximately equivalent to that of white males. Cancer incidence rates for white males do not differ radically from those for other racial groups. The major sites of cancer are the lung and prostate; other important cancer sites among white males are the colon and bladder.

These data confirm the unusually high incidence of nasopharyngeal cancer among Chinese males which has been extensively reported (Buell, 1965; Clifford, 1970; Muir, 1967; Zippin *et al.*, 1962). Chinese males also appear to be at greater risk of developing cancer of the colon than males of other racial groups.

The age-adjusted incidence rate of 50/100,000 for Japanese males is the highest stomach cancer incidence found among the major racial groups studied; stomach cancer accounts for one out of every 4 cancer cases among Japanese males in Alameda County. Japanese males also have the lowest incidence of lung cancer; their age-adjusted incidence rate of 20 is less than one-half that of Chinese males, the group with the next lowest incidence.

Of particular note among Chinese females are the breast cancer incidence of 73/100,000, which is essentially equivalent to that for white females, and the unusually high incidence of lung cancer. In view of the high male Chinese incidence of cancer of the colon, a curious result is that Chinese females have the lowest colon cancer incidence of the racial groups analysed.

Japanese women, like their male

TABLE III.—*Age-adjusted Cancer Incidence Rates, Selected Sites by Sex and Race, Alameda County, California,* 1960–69

| Primary site | White | Negro | Chinese | Japanese |
|---|---|---|---|---|
| | Male | | | |
| All sites | 287·5 | 297·9 | 239·3† | 192·0* |
| Nasopharynx | 0·8 | 0·6 | 15·0† | 0·0 |
| Stomach | 14·5 | 22·9* | 9·9 | 50·4† |
| Colon | 26·5 | 18·7* | 32·1 | 14·8‡ |
| Rectum and anus | 16·6 | 9·3* | 14·6 | 14·0 |
| Pancreas | 10·0 | 13·9† | 9·8 | 14·8 |
| Bronchus and lung | 54·1 | 57·0 | 50·4 | 19·8* |
| Prostate | 44·4 | 80·4* | 17·0* | 16·5* |
| Bladder | 20·7 | 10·0* | 10·6† | 16·0 |
| Leukaemia | 9·7 | 10·1 | 6·0 | 4·4 |
| | Female | | | |
| All sites | 258·8 | 227·1* | 238·4 | 264·4 |
| Stomach | 7·6 | 8·2 | 9·3 | 56·6* |
| Colon | 25·3 | 18·9† | 17·0 | 36·5 |
| Rectum and anus | 10·8 | 10·2 | 11·0 | 4·3 |
| Pancreas | 6·3 | 8·5‡ | 0·0 | 4·2 |
| Bronchus and lung | 11·1 | 11·8 | 18·7 | 12·4 |
| Breast | 73·8 | 52·0* | 73·2 | 43·9† |
| Cervix uteri | 19·0 | 34·8* | 19·2 | 22·2 |
| Corpus uteri | 22·2 | 17·5† | 14·3 | 12·6 |
| Ovary | 13·3 | 10·1† | 5·1† | 1·9* |
| Leukaemia | 6·7 | 4·2† | 13·4 | 10·8 |

* $P \leq 0.001$.
† $0{\cdot}001 < P \leq 0{\cdot}05$.
‡ $0{\cdot}05 < P \leq 0{\cdot}10$.
Rates are per 100,000 population.
Excludes *in situ* cases and basal and squamous cell carcinoma of the skin.
The symbols *, † and ‡ represent the level of significance of the difference between the rate and the corresponding white rate.

counterparts, also have a much higher stomach cancer incidence than white, negro or Chinese women. They also differ from other women in having low breast cancer rates. There are indications, however, of a rising incidence of breast cancer among Japanese women in California. A preliminary analysis of notifications from the San Francisco Bay area section of the Third National Cancer Survey, based on incomplete reporting for 1969–71, reveals that breast cancer is responsible for 36·2% of all cancers reported among California Japanese women under the age of 55 (Japanese Cancer Project, 1970). Data for the native Japanese population are available from Miyagi Prefecture; for the period 1962–64, 14·4% of all cancers occurring in female residents of Miyagi Prefecture under the age of 55 were attributable to breast cancer (Segi, 1970). The comparable figure for white women in Alameda County, California, 1960–69, is 33·6. Although based on small numbers, Alameda County Cancer Registry trend data for the period 1960–69 suggest a steady increase in the incidence of breast cancer among Japanese women.

Among the California Japanese women, the Nisei (first American born generation) may be registering the greatest proportion of the increase in breast cancer incidence. A further analysis of the Third National Cancer Survey data referred to in the preceding paragraph shows a breast cancer frequency of 11 out of 22 total cancers reported among Nisei women under the age of 55, while for Issei (the immigrant generation) women, the corresponding figures are 6 and 25.

White women have the highest incidence of breast cancer and negro women have the highest incidence of cervical cancer. With these 2 exceptions, however, the age-adjusted cancer incidence rates for these two racial groups are similar for the sites considered in this report.

The extent to which socio-economic factors are responsible for the observed differences in breast cancer incidence between white and negro females has been investigated by Zippin and Petrakis (1971). Using Alameda County Cancer Registry data for the period 1960–67, they found that the calculated breast cancer incidence for negro women is greater than for whites at the 3 higher median family income quartiles and is almost identical to that for whites at the lowest quartile. This suggests that at least part of the observed differential in breast cancer incidence may be due to socio-economic factors. Since the above analysis was based on social class data derived from the 1960 census, it is subject to confirmation when comparable 1970 census data become available.

*Trends in cancer incidence*

The relatively small Chinese and Japanese populations of Alameda County, with the resulting small number of cancer cases reported to the Alameda County Cancer Registry, render it undesirable to sub-divide further the available data on Chinese and Japanese cancer cases. For this reason, the trend analysis is limited to whites, negroes and all races combined.

Table IV shows the trend in the age-adjusted incidence of cancer in Alameda County during the period 1960–69. The most notable finding is the consistently upward trend in cancer incidence for all sites combined for both males and females.

Most outstanding in the male data are the increases in cancer of the lung and prostate between 1960 and 1969. The incidence of stomach cancer, on the other hand, has declined. The increase in lung cancer is almost certainly primarily the result of the history of long exposure to cigarette smoking in those men who are now in the age group where lung cancer most frequently occurs.

TABLE IV.—*Trends in Age-adjusted Cancer Incidence Rates, Selected Sites by Sex, Alameda County, California*, 1960–69

| Primary site | 1960–62 | 1963–65 | 1966–67 | 1968–69 |
|---|---|---|---|---|
| | | Male | | |
| All sites | 267·7 | 284·6 | 299·5 | 308·6 |
| Stomach | 17·7 | 16·5 | 12·8 | 14·0 |
| Colon | 24·5 | 25·7 | 25·5 | 28·5 |
| Rectum and anus | 16·4 | 15·3 | 17·8 | 14·1 |
| Pancreas | 9·1 | 10·6 | 10·6 | 11·9 |
| Bronchus and lung | 48·5 | 51·9 | 60·3 | 61·4 |
| Prostate | 40·6 | 48·4 | 51·1 | 49·6 |
| Bladder | 19·7 | 18·2 | 22·5 | 18·7 |
| Leukaemia | 9·9 | 9·4 | 8·8 | 10·4 |
| | | Female | | |
| All sites | 246·4 | 247·5 | 266·0 | 275·0 |
| Stomach | 8·4 | 8·5 | 8·1 | 6·5 |
| Colon | 25·4 | 24·6 | 24·7 | 25·2 |
| Rectum and anus | 11·3 | 10·3 | 11·2 | 10·8 |
| Pancreas | 6·0 | 6·4 | 6·6 | 7·4 |
| Bronchus and lung | 7·7 | 9·8 | 12·0 | 17·8 |
| Breast | 66·4 | 68·5 | 74·6 | 81·1 |
| Cervix uteri | 22·7 | 20·5 | 21·7 | 17·3 |
| Corpus uteri | 18·9 | 18·7 | 25·1 | 26·2 |
| Ovary | 14·0 | 11·4 | 12·3 | 13·8 |
| Leukaemia | 6·2 | 7·2 | 6·6 | 6·3 |

Rates are per 100,000 population. Excludes *in situ* cases and basal and squamous cell carcinoma of the skin.

FIG. 1.—Trends in age-adjusted cancer incidence rates for selected sites (1960–69). White males.

The increase in the female age-adjusted cancer incidence rate for all sites combined is attributable primarily to increases in cancer of the lung, breast and corpus uteri. The incidence of lung cancer is much lower in women than in men, but the risk for women is increasing at a much higher rate than the corresponding rate for men. This confirms an earlier finding, based on California mortality, that the rate of increase in lung cancer since 1960 is greater for women than for men (Linden, 1966). The increase in breast cancer may be an artefact of mammography, other examination procedures and educational campaigns promoting self-examination, resulting in the diagnosis of breast cancer that otherwise would not have been detected until later in the course of the disease. The incidence of stomach cancer among females is declining at approximately the same rate as it is among males. The abrupt rise in the incidence of uterine corpus cancer between the middle 2 time periods is a peculiar facet of the female trend data; we are not able at this time to account satisfactorily for this change.

Fig. 1–4 illustrate the trends in cancer incidence among whites and negroes for selected sites during the period 1960–69; these data reveal a general increase in cancer incidence for both groups.

Among the sites analysed for white males, only the rates for stomach cancer, cancer of the rectum and anus, and leukaemia have not increased; the upward trends are statistically significant for colon ($P < 0{\cdot}05$), pancreas ($P < 0{\cdot}05$) lung ($P < 0{\cdot}01$) and prostate ($P < 0{\cdot}01$); the downward trend is statistically significant only for stomach cancer ($P < 0{\cdot}01$).

FIG. 2.—Trends in age-adjusted cancer incidence rates for selected sites (1960–69). White females.

White females exhibit statistically significant upward trends for cancer of the lung ($P < 0{\cdot}01$), breast ($P < 0{\cdot}01$) and corpus uteri ($P < 0{\cdot}01$); the downward trends for stomach cancer ($P < 0{\cdot}05$) and cancer of the cervix uteri ($P < 0{\cdot}01$) are statistically significant. Analysis of the data on an age-specific basis reveals that the increase in breast cancer incidence is primarily confined to women over the age of 65, so oral contraceptive agents are not implicated. The increase in corpus cancer between 1963–65 and 1966–67 previously referred to is limited entirely to white females. The other sites considered in this report demonstrate either very little increase or no trend whatsoever.

The large variation to which the negro rates are subject does not permit any definitive statements regarding the statistical significance of the observed trends. Cancer of the rectum and anus among negro males has doubled over the time period covered by this report. The trends for the remaining sites, except for stomach, prostate and bladder, are also upward. Breast cancer incidence is increasing steadily among negro females; the most spectacular increase, however, is recorded for lung cancer which increased from 5·4/100,000 in 1960–62 to 21·5/100,000 in 1968–69.

*Comparison with the Connecticut Tumour Registry experience*

The question of whether the Alameda County trends are regional or reflect general changes underway throughout the country can be investigated by examining results obtained by other population-based cancer registries. We are fortunate to have Connecticut Tumour Registry age-adjusted cancer incidence trend

FIG. 3.—Trends in age-adjusted cancer incidence rates for selected sites (1960–69). Negro males.

TABLE V.—*Trends in Age-adjusted Cancer Incidence Rates, Selected Sites by Sex, Connecticut,* 1960–69

| Primary site | 1960 | 1961 | 1962 | 1963 | 1964 | 1965 | 1966 | 1967 | 1968 | 1969 |
|---|---|---|---|---|---|---|---|---|---|---|
| | | | | | Male | | | | | |
| Stomach | 22·3 | 18·9 | 19·8 | 16·8 | 17·2 | 16·3 | 16·9 | 13·7 | 14·5 | 15·1 |
| Colon | 29·3 | 28·9 | 29·5 | 29·6 | 32·7 | 29·6 | 31·4 | 30·5 | 30·5 | 30·7 |
| Rectum | 20·0 | 15·9 | 16·0 | 19·5 | 17·3 | 18·6 | 19·4 | 18·8 | 19·4 | 18·4 |
| Pancreas | 11·4 | 9·6 | 9·6 | 9·8 | 8·9 | 8·9 | 10·0 | 10·1 | 9·2 | 10·2 |
| Bronchus and lung | 47·0 | 46·5 | 46·4 | 47·7 | 47·3 | 50·4 | 54·2 | 50·5 | 52·8 | 56·5 |
| Prostate | 42·5 | 38·8 | 36·8 | 37·1 | 38·3 | 38·5 | 39·0 | 39·2 | 41·0 | 39·5 |
| Bladder | 21·5 | 18·2 | 18·8 | 19·3 | 23·5 | 24·7 | 24·3 | 23·2 | 21·6 | 21·8 |
| Leukaemia | 11·5 | 10·6 | 10·0 | 11·0 | 10·7 | 9·1 | 9·2 | 10·2 | 9·8 | 9·8 |
| | | | | | Female | | | | | |
| Stomach | 10·6 | 10·1 | 9·6 | 8·4 | 7·5 | 7·8 | 6·8 | 7·5 | 6·1 | 6·5 |
| Colon | 27·9 | 28·5 | 28·7 | 30·4 | 32·2 | 28·6 | 28·7 | 29·4 | 29·0 | 30·5 |
| Rectum | 12·5 | 11·1 | 11·3 | 13·6 | 10·9 | 11·8 | 14·6 | 11·7 | 11·5 | 13·2 |
| Pancreas | 6·4 | 5·9 | 6·6 | 4·9 | 5·5 | 4·6 | 5·5 | 6·8 | 6·6 | 6·4 |
| Bronchus and lung | 6·4 | 8·0 | 6·7 | 7·7 | 8·2 | 9·4 | 7·0 | 10·5 | 11·7 | 11·2 |
| Breast | 64·9 | 70·0 | 64·3 | 64·5 | 68·5 | 75·1 | 74·9 | 68·6 | 71·0 | 80·9 |
| Corpus uteri | 14·4 | 15·8 | 14·0 | 17·6 | 16·5 | 17·2 | 16·6 | 17·7 | 18·2 | 17·6 |
| Ovary | 13·8 | 15·0 | 13·7 | 12·7 | 11·5 | 13·3 | 12·1 | 13·3 | 13·3 | 14·6 |
| Bladder | 6·4 | 5·3 | 5·8 | 6·4 | 6·9 | 6·9 | 6·1 | 6·2 | 6·2 | 6·0 |
| Leukaemia | 6·6 | 6·3 | 6·7 | 7·4 | 6·7 | 5·4 | 6·2 | 6·7 | 5·8 | 5·7 |

Rates are per 100,000 population. Rates are age-adjusted to the U.S. 1950 population.
Source: State of Connecticut, Department of Health, Connecticut Tumour Registry, unpublished data.

FIG. 4.—Trends in age-adjusted cancer incidence rates for selected sites (1960–69). Negro females.

data for the period 1960–69 available for this purpose, since similar coding systems and methodologies for the derivation of cancer incidence data are used by the Connecticut and Alameda County population-based registries. The population of Connecticut is almost entirely white; data for only the white population of Alameda County are therefore employed in the comparison. The Connecticut trend data are shown in Table V; the results of the comparison are outlined in Table VI.

TABLE VI.—*Comparison of Cancer Incidence Data from the Connecticut and Alameda Population-Based Registries—White Males and Females*

| Primary site | Connecticut trend, 1960–69 | Alameda County trend, 1960–69 |
|---|---|---|
| Stomach | Declining among both males and females | Declining among both males and females |
| Colon | No consistent trend | Increasing in males |
| Rectum | Rates are essentially stable | No consistent trend |
| Pancreas | Rates are essentially stable | Moderate increase in both sexes |
| Bronchus and lung | Increasing among both males and females | Increasing in both sexes at a higher rate than in Connecticut |
| Breast | Trend is upward | Increasing steadily |
| Corpus uteri | Trend is generally upward | Increase in incidence in recent years |
| Ovary | Rates are essentially stable | No consistent trend |
| Prostate | Rates are essentially stable | Definite increase in incidence |
| Bladder | Rates are essentially stable | Male rates are essentially stable. Female rates are declining |
| Leukaemia | Slight downward trend among sexes | No consistent trend |

DISCUSSION

These results indicate the type of data which can be produced by a population-based cancer registry. They also serve to point out a weakness in the conventional method of registry reporting. Population-based registries in existence today suffer from many delay factors in obtaining data for current incidence. Delays of 1–2 years in the preparation of incidence rates are not uncommon.

The widespread use of various chemical agents and therapeutic procedures and the increasing concentration of environmental contaminants, without information regarding the carcinogenic effect of these agents on humans, make essential the development of data systems that can detect, with the least possible delay, changes in cancer incidence (Fraumeni and Miller, 1972). While it is generally agreed that there is a long latent interval between exposure to a carcinogen and the development of malignancy, earlier detection of changes in cancer incidence will permit the prompt initiation of studies into the factors responsible.

Plans for the development of an immediate notification system which will permit the preparation of preliminary incidence rates within a few months after the completion of a given calender year are currently underway by the California Tumour Registry. Such a procedure will employ a simple reporting form and, with rapid computer based updating, record linkage, census tracting and population estimation techniques, will create a cancer incidence system with an enhanced ability to monitor alterations in the incidence of cancer in the population.

Supported in part by Contract NIH-69-5, Biometry Branch, National Cancer Institute.

REFERENCES

BUELL, P. (1965) Nasopharynx Cancer in Chinese of California. *Br. J. Cancer*, **19**, 459.

CALIFORNIA TUMOUR REGISTRY (1967) Incidence of Cancer in Alameda County, California, 1960–64. State of California Department of Public Health.

CLIFFORD, P. (1970) On the Epidemiology of Nasopharyngeal Carcinoma. *Int. J. Cancer*, **5**, 287.

FRAUMENI, J. F. & MILLER, R. W. (1972) Drug-Induced Cancer. *J. natn. Cancer Inst.*, **48**, 1267.

JAPANESE CANCER PROJECT (1970) Project Records, San Francisco Bay Area Resource for Cancer Epidemiology. State of California Department of Public Health.

LINDEN, G. (1966) The Increasing Rate of Lung Cancer Mortality in California Women. *Calif. Sch. Hlth*, **2**, 12.

MUIR, C. S. (1967) Nasopharyngeal Carcinoma; A Historical Vignette. In *Cancer of the Nasopharynx*. Ed. C. S. Muir and K. Shanmagaratnam. UICC Monograph Series, **1**, 47.

SEGI, M. (1970) Japan, Miyagi Prefecture. In *Cancer Incidence in Five Continents*, Vol. II. Ed. R. Doll, C. S. Muir and J. A. H. Waterhouse. Geneva: UICC.

ZIPPIN, C., TEKAWA, I., BRAGG, K., WATSON, D. & LINDEN, G. (1962) Studies on Heredity and Environment in Cancer of the Nasopharynx. *J. natn. Cancer Inst.*, **29**, 483.

ZIPPIN, C. & PETRAKIS, N. L. (1971) Identification of High Risk Groups in Breast Cancer. *Cancer, N.Y.*, **28**, 1381.